# Applications of Machine Learning in Cancer Prediction and Prognosis

**Published:** 2007-02-11

**Authors:** Joseph A. Cruz, David S. Wishart

**Affiliations:** Departments of Biological Science and Computing Science, University of Alberta Edmonton, AB, Canada T6G 2E8

**Keywords:** Cancer, machine learning, prognosis, risk, prediction

## Abstract

Machine learning is a branch of artificial intelligence that employs a variety of statistical, probabilistic and optimization techniques that allows computers to “learn” from past examples and to detect hard-to-discern patterns from large, noisy or complex data sets. This capability is particularly well-suited to medical applications, especially those that depend on complex proteomic and genomic measurements. As a result, machine learning is frequently used in cancer diagnosis and detection. More recently machine learning has been applied to cancer prognosis and prediction. This latter approach is particularly interesting as it is part of a growing trend towards personalized, predictive medicine. In assembling this review we conducted a broad survey of the different types of machine learning methods being used, the types of data being integrated and the performance of these methods in cancer prediction and prognosis. A number of trends are noted, including a growing dependence on protein biomarkers and microarray data, a strong bias towards applications in prostate and breast cancer, and a heavy reliance on “older” technologies such artificial neural networks (ANNs) instead of more recently developed or more easily interpretable machine learning methods. A number of published studies also appear to lack an appropriate level of validation or testing. Among the better designed and validated studies it is clear that machine learning methods can be used to substantially (15–25%) improve the accuracy of predicting cancer susceptibility, recurrence and mortality. At a more fundamental level, it is also evident that machine learning is also helping to improve our basic understanding of cancer development and progression.

## Introduction

Machine learning is not new to cancer research. Artificial neural networks (ANNs) and decision trees (DTs) have been used in cancer detection and diagnosis for nearly 20 years (Simes 1985; Maclin et al. 1991; Ciccheti 1992). Today machine learning methods are being used in a wide range of applications ranging from detecting and classifying tumors via X-ray and CRT images (Petricoin and Liotta 2004; Bocchi et al. 2004) to the classification of malignancies from proteomic and genomic (microarray) assays (Zhou et al. 2004; Dettling 2004; Wang et al. 2005). According to the latest PubMed statistics, more than 1500 papers have been published on the subject of machine learning and cancer. However, the vast majority of these papers are concerned with using machine learning methods to identify, classify, detect, or distinguish tumors and other malignancies. In other words machine learning has been used primarily as an aid to cancer diagnosis and detection (McCarthy et al. 2004). It has only been relatively recently that cancer researchers have attempted to apply machine learning towards cancer prediction and prognosis. As a consequence the body of literature in the field of machine learning and cancer prediction/prognosis is relatively small (<120 papers).

The fundamental goals of cancer prediction and prognosis are distinct from the goals of cancer detection and diagnosis. In cancer prediction/prognosis one is concerned with three predictive foci: 1) the prediction of cancer susceptibility (i.e. risk assessment); 2) the prediction of cancer recurrence and 3) the prediction of cancer survivability. In the first case, one is trying to predict the likelihood of developing a type of cancer prior to the occurrence of the disease. In the second case one is trying to predict the likelihood of redeveloping cancer after to the apparent resolution of the disease. In the third case one is trying to predict an outcome (life expectancy, survivability, progression, tumor-drug sensitivity) after the diagnosis of the disease. In the latter two situations the success of the prognostic prediction is obviously dependent, in part, on the success or quality of the diagnosis. However a disease prognosis can only come after a medical diagnosis and a prognostic prediction must take into account more than just a simple diagnosis (Hagerty et al. 2005).

Indeed, a cancer prognosis typically involves multiple physicians from different specialties using different subsets of biomarkers and multiple clinical factors, including the age and general health of the patient, the location and type of cancer, as well as the grade and size of the tumor (Fielding et al. 1992; Cochran 1997; Burke et al. 2005). Typically histological (cell-based), clinical (patient-based) and demographic (population-based) information must all be carefully integrated by the attending physician to come up with a reasonable prognosis. Even for the most skilled clinician, this is not easy to do. Similar challenges also exist for both physicians and patients alike when it comes to the issues of cancer prevention and cancer susceptibility prediction. Family history, age, diet, weight (obesity), high-risk habits (smoking, heavy drinking), and exposure to environmental carcinogens (UV radiation, radon, asbestos, PCBs) all play a role in predicting an individual’s risk for developing cancer (Leenhouts 1999; Bach et al. 2003; Gascon et al. 2004; Claus 2001; Domchek et al. 2003). Unfortunately these conventional “macro-scale” clinical, environmental and behavioral parameters generally do not provide enough information to make robust predictions or prognoses. Ideally what is needed is some very specific molecular details about either the tumor or the patient’s own genetic make-up (Colozza et al. 2005).

With the rapid development of genomic (DNA sequencing, microarrays), proteomic (protein chips, tissue arrays, immuno-histology) and imaging (fMRI, PET, micro-CT) technologies, this kind of molecular-scale information about patients or tumors can now be readily acquired. Molecular biomarkers, such as somatic mutations in certain genes (p53, BRCA1, BRCA2), the appearance or expression of certain tumor proteins (MUC1, HER2, PSA) or the chemical environment of the tumor (anoxic, hypoxic) have been shown to serve as very powerful prognostic or predictive indicators (Piccart et al. 2001; Duffy 2001; Baldus et al. 2004). More recently, combinations or patterns of multiple molecular biomarkers have been found to be even more predictive than single component tests or readouts (Savage and Gascoyne 2004; Petricoin and Liotta 2004; Duffy 2005; Vendrell et al. 2005) If these molecular patterns are combined with macro-scale clinical data (tumor type, hereditary aspects, risk factors), the robustness and accuracy of cancer prognoses and predictions improves even more. However, as the number of parameters we measure grows, so too does the challenge of trying to make sense of all this information.

In the past, our dependency on macro-scale information (tumor, patient, population, and environmental data) generally kept the numbers of variables small enough so that standard statistical methods or even a physician’s own intuition could be used to predict cancer risks and outcomes. However, with today’s high-throughput diagnostic and imaging technologies we now find ourselves overwhelmed with dozens or even hundreds of molecular, cellular and clinical parameters. In these situations, human intuition and standard statistics don’t generally work. Instead we must increasingly rely on non-traditional, intensively computational approaches such as machine learning. The use of computers (and machine learning) in disease prediction and prognosis is part of a growing trend towards personalized, predictive medicine (Weston and Hood 2004). This movement towards predictive medicine is important, not only for patients (in terms of lifestyle and quality-of-life decisions) but also for physicians (in making treatment decisions) as well as health economists and policy planners (in implementing large scale cancer prevention or cancer treatment policies).

**Figure 1.**
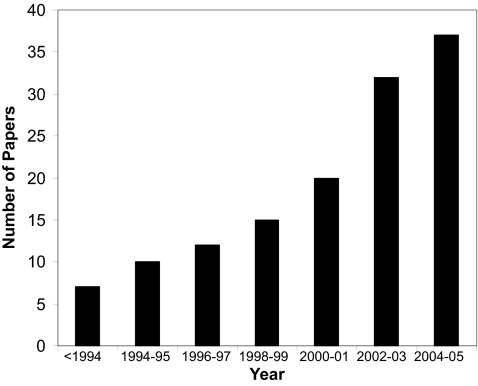
A histogram showing the steady increase in published papers using machine learning methods to predict cancer risk, recurrence and outcome. The data were collected using a variety of keyword searches through PubMed, CiteSeer, Google Scholar, Science Citation Index and other online resources. Each bar represents the cumulative total of papers published over a two year period. The earliest papers appeared in the early 1990’s.

Given the growing importance of predictive medicine and the growing reliance on machine learning to make predictions, we believed it would be of interest to conduct a detailed review of published studies employing machine learning methods in cancer prediction and prognosis. The intent is to identify key trends with respect to the types of machine learning methods being used, the types of training data being integrated, the kinds of endpoint predictions being made, the types of cancers being studied and the overall performance of these methods in predicting cancer susceptibility or patient outcomes. Interestingly, when referring to cancer prediction and prognosis we found that most studies were concerned with three “predictive” foci or clinical endpoints: 1) the prediction of cancer susceptibility (i.e. risk assessment); 2) the prediction of cancer recurrence and 3) the prediction of cancer survivability. We also found that almost all predictions are made using just four types of input data: genomic data (SNPs, mutations, microarrays), proteomic data (specific protein biomarkers, 2D gel data, mass spectral analyses), clinical data (histology, tumor staging, tumor size, age, weight, risk behavior, etc.) or combinations of these three. In comparing and evaluating the existing studies a number of general trends were noted and a number of common problems detected. Some of the more obvious trends include a rapidly growing use of machine learning methods in cancer prediction and prognosis (Figure 1), a growing reliance on protein markers and microarray data, a trend towards using mixed (proteomic + clinical) data, a strong bias towards applications in prostate and breast cancer, and an unexpected dependency on older technologies such as artificial neural networks (ANNs). Among the more commonly noted problems was an imbalance of predictive events with parameters (too few events, too many parameters), overtraining, and a lack of external validation or testing. Nevertheless, among the better designed and better validated studies it was clear that machine learning methods, relative to simple statistical methods, could substantially (15–25%) improve the accuracy of cancer susceptibility and cancer outcome prediction. In other words, machine learning has an important role to play in cancer prediction and prognosis.

## Machine Learning Methods

Before beginning with a detailed analysis of what machine learning methods work best for which kinds of situations, it is important to have a good understanding of what machine learning is – and what it isn’t. Machine learning is a branch of artificial intelligence research that employs a variety of statistical, probabilistic and optimization tools to “learn” from past examples and to then use that prior training to classify new data, identify new patterns or predict novel trends (Mitchell 1997). Machine learning, like statistics, is used to analyze and interpret data. Unlike statistics, though, machine learning methods can employ Boolean logic (AND, OR, NOT), absolute conditionality (IF, THEN, ELSE), conditional probabilities (the probability of X given Y) and unconventional optimization strategies to model data or classify patterns. These latter methods actually resemble the approaches humans typically use to learn and classify. Machine learning still draws heavily from statistics and probability, but it is fundamentally more powerful because it allows inferences or decisions to be made that could not otherwise be made using conventional statistical methodologies (Mitchell 1997; Duda et al. 2001). For instance, many statistical methods are based on multivariate regression or correlation analysis. While generally very powerful, these approaches assume that the variables are independent and that data can be modeled using linear combinations of these variables. When the relationships are nonlinear and the variables are interdependent (or conditionally dependent) conventional statistics usually flounders. It is in these situations where machine learning tends to shine. Many biological systems are fundamentally nonlinear and their parameters conditionally dependent. Many simple physical systems are linear and their parameters are essentially independent.

Success in machine learning is not always guaranteed. As with any method, a good understanding of the problem and an appreciation of the limitations of the data is important. So too is an understanding of the assumptions and limitations of the algorithms being applied. If a machine learning experiment is properly designed, the learners correctly implemented and the results robustly validated, then one usually has a good chance at success. Obviously if the data is of poor quality, the result will be of poor quality (garbage in = garbage out). Likewise if there are more variables than events to predict then it is also possible to create a series of redundant learners. This is a set of learning algorithms that seems to perform at the same (low) level regardless of the choice of input data. The problem of too many variables and too few examples is called the “curse of dimensionality” (Bellman 1961). This curse is not restricted to machine learning. It also affects many statistical methods as well. The only solution is to reduce the number of variables (features) or increase the number of training examples. As a general rule, the sample-per-feature ratio should always exceed 5:1 (Somorjai et al. 2003). Not only is the size of the training set important, so too is the variety of the training set. Training examples should be selected to span a representative portion of the data the learner expects to encounter. Training too many times on too few examples with too little variety leads to the phenomenon of over-training or simply training on noise (Rodvold et al. 2001). An over-trained learner, just like an overtired student, will generally perform poorly when it tries to process or classify novel data.

Sometimes conventional statistics proves to be more powerful or more accurate than machine learning. In these casesthe user's initial determinations about the interdependence and nonlinearity of the data would have been wrong. This is not necessarily a weakness to machine learning, it is just a matter of choosing the right tool for the right job. Likewise, not all machine learning methods are created equal. Some are better for certain kinds of problems while others are better for other kinds of problems. For instance some machine learning algorithms scale nicely to the size of the biological domains, others do not. Likewise some methods may have assumptions or data requirements that render them inapplicable to the problem at hand. Knowing which method is best for a given problem is not inherently obvious. This is why it is critically important to try more than one machine learning method on any given training set. Another common misunderstanding about machine learning is that the patterns a machine learning tool finds or the trends it detects are non-obvious or not intrinsically detectable. On the contrary, many patterns or trends could be detected by a human expert – if they looked hard enough at the data. Machine learning simply saves on the time and effort needed to discover the pattern or to develop the classification scheme. Recall that with any interesting discovery, it is frequently obvious to the casual observer – particularly after the discovery has been made.

**Figure 2.**
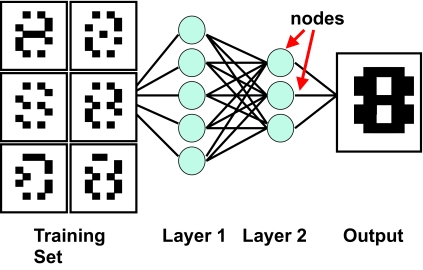
An example of how a machine learner is trained to recognize images using a training set (a corrupted image of the number “8”) which is labeled or identified as the number “8”.

There are three general types of machine learning algorithms: 1) supervised learning; 2) unsupervised learning and 3) reinforcement learning. They are essentially classified on the basis of desired outcome of the algorithm (Mitchell, 1997; Duda et al. 2001). In supervised learning algorithms a “prescient provider” or teacher gives the learning algorithm a labeled set of training data or examples. These labeled examples are the training set that the program tries to learn about or to learn how to map the input data to the desired output. For instance a labeled training set might be a set of corrupted images of the number “8” (Figure 2). Since all the images are labeled as being the number “8” and the desired output is the uncorrupted “8”, the learner is able to train under the supervision of a teacher telling it what it is supposed to find. This is the process by which most school children learn. In unsupervised learning, a set of examples are given, but no labels are provided. Instead it is up to the learner to find the pattern or discover the groups. This is somewhat analogous to the process by which most graduate students learn. Unsupervised learning algorithms include such methods as self-organizing feature maps (SOMs), hierarchical clustering and K-means clustering algorithms. These approaches create clusters from raw, unlabeled or unclassified data. These clusters can be used later to develop classification schemes or classifiers.

The SOM approach (Kohonen 1982) is a specialized form of a neural network or ANN. It is based on using a grid of artificial neurons whose weights are adapted to match input vectors in a training set. In fact, the SOM was originally designed to model biological brain function (Kohonen 1982). A SOM begins with a set of artificial neurons, each havingits own physical location on the output map, which take part in a winner-take-all process (a competitive network) where a node with its weight vector closest to the vector of inputs is declared the winner and its weights are adjusted making them closer to the input vector. Each node has a set of neighbors. When this node wins a competition, the neighbors’ weights are also changed, albeit to a lesser extent. The further the neighbor is from the winner, the smaller its weight change. This process is then repeated for each input vector for a large number of cycles. Different inputs produce different winners. The net result is a SOM which is capable of associating output nodes with specific groups or patterns in the input data set.

Interestingly, almost all machine learning algorithms used in cancer prediction and prognosis employ supervised learning. Furthermore, most of these supervised learning algorithms belong to a specific category of classifiers that classify on the basis of conditional probabilities or conditional decisions. The major types of conditional algorithms include: 1) artificial neural networks (ANN – Rummelhart et al. 1986); 2) decision trees (DT – Quinlan, 1986); 3) genetic algorithms (GA – Holland 1975); 4) linear discriminant analysis (LDA) methods; 5) *k*-nearest neighbor algorithms prognosis with more than 820 of 1585 surveyed papers using or referring to ANNs. First developed by McCulloch and Pitts (1943) and later popularized in the 1980’s by Rumelhart et al. (1986), ANNs are capable of handling a wide range of classification or pattern recognition problems. Their strength lies in being able to perform a range of statistical (linear, logistic and nonlinear regression) and logical operations or inferences (AND, OR, XOR, NOT, IF-THEN) as part of the classification process (Rodvold et al. 2001; Mitchell 1997). ANNs were originally designed to model the way the brain works with multiple neurons being interconnected to each other through multiple axon junctions. Just as with biological learning, the strength of the neural connections is strengthened or weakened through repeated training or reinforcement on labeled training data. Mathematically, these neural connections can be represented as a wiring table or matrix (i.e. neuron 1 is connected to neuron 2, 4 and 7; neuron 2 is connected to neuron 1, 5, 6 and 8, etc.). This weight matrix is called a layer, in analogy to the cortical layers in the brain. Neural networks typically use multiple layers (called hidden layers) to process their input and generate an output (Figure 2). To comply with the mathematical structure of each layer, input and output data is normally structured as a string, or vector, of numbers. One of the challenges in using ANNs is mapping how the real-world input/output (an image, a physical characteristic, a list of gene names, a prognosis) can be mapped to a numeric vector. In ANNs the adjustment of neural connection strengths is usually done via an optimization technique called back-propagation (short for backwards propagation of errors – Rumelhart et al. 1986). This is a derivative-based process that compares the output of one layer to the preceding layer’s table. In very simple terms the answers or labeled training data are used to progressively modify the numbers in the neural network’s weight matrices. A learning or information-transfer function (usually a sigmoidal curve) that is easily differentiable is required for back propagation. Most ANNs are structured using a multi-layered feedforward architecture, meaning they have no feedback, or no connections that loop. The design and structure of an ANN must be customized or optimized for each application. Simply choosing a generic ANN architecture or naively structuring a generic input/output schema can lead to very poor performance or extremely slow training. Another disadvantage of ANNs is the fact that they are a “black-box” technology. Trying to figure out why an ANN didn’t work or how it performs its classification is almost impossible to discern. In other words, the logic of a trained ANN is not easy to decipher.

**Figure 3.**
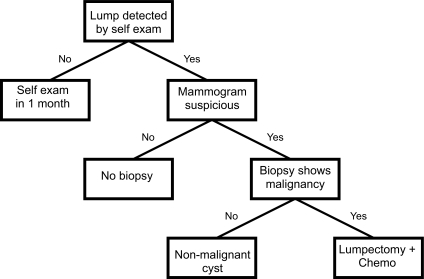
An example of a simple decision tree that might be used in breast cancer diagnosis and treatment. This is an example of a tree that might be formulated via expert assessment. Similar tree structures can be generated by decision tree learners.

In contrast to ANNs, the logic of decision trees (DTs) is very easy to discern. Formally a decision tree is a structured graph or flow chart of decisions (nodes) and their possible consequences (leaves or branches) used to create a plan to reach a goal (Quinlan, 1986; Mitchell 1997). Decision trees have been around for centuries (especially in taxonomy) and are a common component to many medical diagnostic protocols. An outline of a simple decision tree for breast cancer diagnosis is given in Figure 3. Normally decision trees are designed through consultation with experts and refined through years of experience or modified to comply with resource limitations or to limit risk. However decision tree learners also exist which can automatically construct decision trees given a labeled set of training data. When decision tree learners are used to classify data the leaves in the tree represent classifications and branches represent conjunctions of features that lead to those classifications. A decision tree can be learned by progressively splitting the labeled training data into subsets based on a numerical or logical test (Quinlan 1986). This process is repeated on each derived subset in a recursive manner until further splitting is either not possible, or a singular classification is achieved. Decision trees have many advantages: they are simple to understand and interpret, they require little data preparation, they can handle many types of data including numeric, nominal (named) and categorical data, they generate robust classifiers, they are quick to “learn” and they can be validated using statistical tests. However DTs do not generally perform as well as ANNs in more complex classification problems (Atlas et al. 1990).

**Figure 4.**
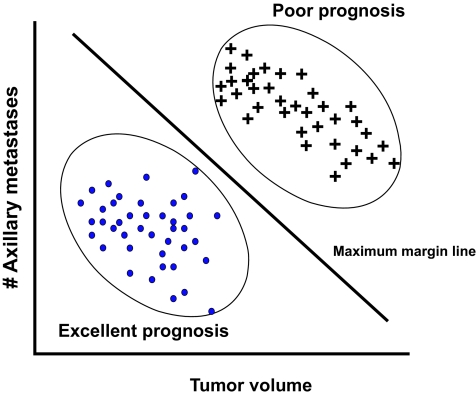
A simplified illustration of how an SVM might work in distinguishing between basketball players and weightlifters using height/weight support vectors. In this simple case the SVM has identified a hyperplane (actually a line) which maximizes the separation between the two clusters.

A somewhat newer machine learning technique is called a support vector machine or SVM (Vapnik, 1982; Cortes and Vapnik 1995; Duda et al. 2001). SVMs are well known in the world of machine learning but almost unknown in the field of cancer prediction and prognosis (see Table 2). How an SVM works can best be understood if one is given a scatter plot of points, say of tumor mass versus number of axillary metastases (for breast cancer) among patients with excellent prognoses and poor prognoses (Figure 4). Two clusters are obviously evident. What the SVM machine learner would do is find the equation for a line that would separate the two clusters maximally. If one was plotting more variables (say volume, metastases and estrogen receptor content) the line of separation would become a plane. If more variables were included the separation would be defined by a hyperplane. The hyperplane is determined by a subset of the points of the two classes, called support vectors. Formally, the SVM algorithm creates a hyperplane that separates the data into two classes with the maximum margin – meaning that the distance between the hyperplane and the closest examples (the margin) is maximized. SVMs can be used to perform nonlinear classification using what is called a non-linear kernel. A non-linear kernel is a mathematical function that transforms the data from a linear feature space to a non-linear feature space. Applying different kernels to different data sets can dramatically improve the performance of an SVM classifier. LikeANNs, SVMs can be used in a wide range of pattern recognition and classification problems ranging from hand writing analysis, speech and text recognition, protein function prediction and medical diagnosis (Duda et al. 2001). SVMs are particularly well suited to non-linear classification problems, as are *k*-nearest neighbor approaches (see Table 1).

## A Survey of Machine Learning Applications in Cancer Prediction

In preparing this review several electronic databases were accessed including PubMed (biomedical literature), the Science Citation Index (biomedical, engineering, computing and physico-chemical literature), CiteSeer (computing literature), Google and Google Scholar (web-accessible scientific literature). Query terms included “cancer and machine learning”, “cancer prediction and machine learning”, “cancer prognosis and machine learning”, “cancer risk assessment and machine learning” as well as multiple sub-queries with specific types of machine learning algorithms. The relevance of the individual papers was assessed by reading the titles and abstracts and identifying papers that used recognizable machine learning methods as well as molecular, clinical, histological, physiological or epidemiological data in carrying out a cancer prognosis or prediction. Papers that focused on diagnoses or simple tumor classifications were excluded as were papers that had coincidental appearances of the words “machine” or “learning” in their abstracts. A PubMed search of “cancer and machine learning” yielded 1585 results, while searches of “cancer prediction and machine learning” and “cancer prognosis and machine learning” yielded 174 and 240 hits respectively. A detailed review of these abstracts led to the identification of 103 relevant papers of which 71 could be accessed through various library holdings. Using CiteSeer, a search with the terms “cancer and machine learning” yielded 349 results, of which 12 (3.4%) were deemed relevant to cancer prognosis. Using Google Scholar, a search using “cancer prognosis and ‘machine learning’” yielded 996 results, of which 49 (4.9%) were judged relevant to cancer prognosis. Many of these papers were previously identified in the PubMed searches as were the vast majority of the hits in the Science Citation Index searches. From the initial group of papers identified from these electronic searches, their reference lists were further consulted to identify additional papers of interest or relevance. In the end more than 120 relevant papers, going as far back as 1989, were identified. Of these, 79 papers could be accessed from existing library holdings and were selected for more detailed analysis (Table 2). While it is impossible to be certain that we achieved complete coverage of all literature on machine learning and cancer prediction/prognosis, we believe that a significant portion of the relevant literature has been assessed for this review.

From our analysis of the literature several trends were noted. As has been remarked previously, the use of machine learning in cancer prediction and prognosis is growing rapidly, with the number of papers increasing by 25% per year (Figure 1). While it is clear that machine learning applications in cancer prediction and prognosis are growing, so too is the use of standard statistically-based predictive methods.

In particular, we looked at the frequency with which “cancer prediction prognosis methods” and “cancer risk assessment prediction methods” occurred in PubMed. These queries yielded 1061 and 157 hits respectively, giving a non-overlapping set of 1174 papers. Removing the 53 papers with machine learning components in this set, we were left with 1121 papers. While a detailed review of each abstract was not possible, a random sampling indicated that ~80% of these papers were relevant (890 papers) in that they used statistical approaches to predict or prognosticate cancer outcomes. Therefore these data suggest that machine learning methods account for 103/890 (11%) of all PubMed papers describing cancer prediction or prognosis methodology. Overall, the same yearly growth trends (i.e. near exponential) in prediction and prognosis were observed for the statistical methods as for the machine learning methods.

When looking at the types of predictions or prognoses being made, the vast majority (86%) are associated with predicting cancer mortality (44%) and cancer recurrence (42%). However, a growing number of more recent studies are now aimed at predicting the occurrence of cancer or the risk factors associated with developing cancer. As a general rule, regardless of the machine learning method used, the type of prediction being made or the type of cancer being evaluated, machine learning methods appear to improve the accuracy of predictions by and average of 15–25% over alternative or conventional approaches (Table 2).

In assessing how these predictions were made it appears that the majority (53%) studies relied on clinical (cancer staging, cellular histology, nuclear markers) or demographic data (age, weight, smoking) – either alone or in combination with other molecular biomarkers. While histological data is generally more accessible, the ambiguity or pathologist-specific peculiarities of many histopathological assessments almost always makes it difficult to generalize or transfer a machine learning tool trained on this kind of data to other clinical settings. Given the limitations of using histological assessments in machine learning, there is an encouraging trend among more recent studies to use more robustly measurable features such as specific protein markers, gene mutations and gene expression values as input data. Approximately 47% of studies used this molecular (i.e. proteomic or genomic) data either alone (25%) or in combination (22%) with clinical data. Given the precision of most molecular assays (with the possible exception of microarray data), we believe the results from these studies should be more easily or robustly transferable to other clinical settings.

**Figure 5.**
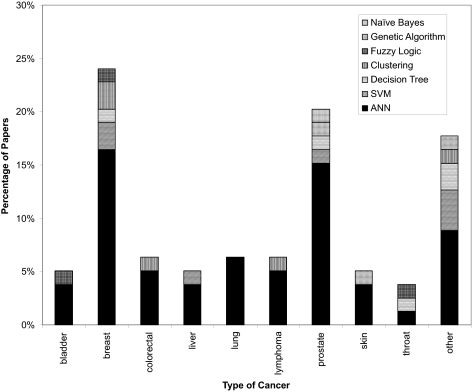
A histogram showing the frequency with which different types of machine learning methods are used to predict different types of cancer. Breast and prostate cancer dominate, however a good range of cancers from different organs or tissues also appear to be compatible with machine learning prognoses. The “other” cancers include brain, cervical, esophageal, leukemia, head, neck, ocular, osteosarcoma, pleural mesothelioma, thoracic, thyroid, and trophoblastic (uterine) malignancies. Figure 1.

As seen in Figure 5, there is strong bias among scientists to use machine learning towards predicting outcomes or risks associated with breast (24%) and prostate (20%) cancer. This, no doubt, reflects the higher frequency of these cancers among patients in Europe and North America. Nevertheless, machine learning methods appear to have been successfully used in predicting outcomes or risks in nearly a dozen different kinds of cancer. This suggests that machine learning methods can be quite generally applied to cancer prediction and prognosis. Figure 5 also illustrates the distribution of the types of machine learning methods applied to different kinds of cancer predictions. Almost 70% of all reported studies use neural networks as their primary (and sometimes only) predictor. Support vector machines are a distant second with 9%, while clustering and decision trees each account for about 6%. Genetic algorithms and other methods (naïve Bayes, fuzzy logic) are rarely used (Table 2). This is both surprising and a bit disappointing. ANNs are relatively old machine learning technologies which yield so-called “black-box” results. That is, their performance and classification processes are not easily explained or rationalized. The existence of other methods (SVMs, DTs, NBs) which inherently provide easily accessible explanations appears not to be widely known among cancer informaticians. Overall, many of the papers reviewed for this survey were of generally high quality. Some of the better papers are discussed in more detail under the “Case Studies” section of this review. However, a disturbing number of studies lacked sufficient internal or external validation, were trained on far too few examples, tested on only a single machine learner or had no well-defined standard with which to compare the performance of the reported algorithm. These problems are discussed in more detail under the section entitled “Limitations and Lessons”.

### Case Study 1 – Cancer Risk or Susceptibility Prediction

Of the 79 papers surveyed in this review, relatively few papers (just 3) employed machine learning to predict cancer risk susceptibility. One of the more interesting papers (Listgarten et al. 2004), used single nucleotide polymorphism (SNP) profiles of steroid metabolizing enzymes (CYP450s) to develop a method to retrospectively predict the occurrence of “spontaneous” breast cancer. Spontaneous or non-familial breast cancer accounts for about 90% of all breast cancers (Dumitrescu and Cotarla 2005). The hypothesis in this study was that certain combinations of steroid-metabolism gene SNPs would lead to the increased accumulation of environmental toxins or hormones in breast tissue leading to a higher risk for breast cancer. The authors collected SNP data (98 SNPs from 45 different cancer-associated genes) for 63 patients with breast cancer and 74 patients without breast cancer (control). Key to the success of this study was the fact that the authors employed several methods to reduce the sample-per-feature ratio and investigated multiple machine learning methods to find an optimal classifier. Specifically, from a starting set of 98 SNPs the authors quickly reduced this set to just 2–3 SNPs that seemed maximally informative. This reduced the sample-per-feature ratio to a respectable 45:1 (for 3 SNPs) and 68:1 (for 2 SNPs) instead of close to 3:2 (had all 98 SNPs been used). This allowed the study to avoid falling victim to the “curse of dimensionality” (Bellman 1961; Somorjai etal. 2003). Once the sample size was reduced, several machine learning techniques were employed including a naïve Bayes model, several decision tree models and a sophisticated support vector machine (SVM). The SVM and naïve Bayes classifiers attained the highest accuracy using only a set of 3 SNPs and the decision tree classifier attained the highest accuracy using a set of 2 SNPs. The SVM classifier performed the best with an accuracy of 69%, while the naïve Bayes and decision tree classifiers achieved accuracies of 67% and 68%, respectively. These results are approximately 23–25% better than chance. Another notable feature to this study was the extensive level of cross validation and confirmation performed. The predictive power of each model was validated in at least three ways. Firstly, the training of the models were assessed and monitored with 20-fold cross-validation. A bootstrap resampling method was employed by performing the cross-validation 5 times and averaging the results so as to minimize the stochastic element involved with partitioning of the samples. Secondly, to minimize the bias in feature selection (i.e. selecting the most informative subset of SNPs), the selection process was performed within each fold for a total of 100 times (5 times for each of the 20 folds). Finally, the results were compared against a random permutation test which at best, had a predictive accuracy of 50%. While the authors attempted to minimize the stochastic element involved with partitioning of the samples, a better method may have been to use leave-one-out cross-validation which would have removed this stochastic element completely. That being said, the multiple cross-validations resulted in a standard deviation that was not more than 4% for any of the reported accuracies and since all the methods performed close to 25% better than chance, this standard deviation is deemed negligible. While no external validation set was reported in this study, we have recently learned that the results described in this paper have been duplicated with a similar follow-on study of another 200 individuals (S. Damaraju, personal communication). Overall, this study nicely illustrates how the proper design, careful implementation, appropriate data selection and thorough validation of multiple machine learners can produce a robust and accurate cancer-risk prediction tool. It also highlights how machine learning can reveal important insights into the biology and polygenic risk factors associated with spontaneous or non-familial breast cancer.

### Case Study 2: Prediction of Cancer Survivability

Nearly half of all machine learning studies on cancer prediction were focused on predicting patient survivability (either 1 year or 5 year survival rates). One paper of particular interest (Futschik et al. 2003) used a hybrid machine learning approach to predict outcomes for patients with diffuse large B-cell lymphoma (DLBCL). Specifically, both clinical and genomic (microarray) data were combined to create a single classifier to predict survival of DLBCL patients. This approach differs somewhat from the study of Listgarten et al. (2004) which only employed genomic (SNP) data in its classifier schema. Futschik et al. hypothesized, correctly, that clinical information could enrich microarray data such that a combined predictor would perform better than a classifier based on either microarray data alone or clinical data alone. In assembling the test and training samples, the authors collected microarray expression data and clinical information for 56 DLBCL patients. The clinical information was obtained from the International Prediction Index (IPI) which consists of a set of risk factors, that when properly assessed, allows patients to be separated into groups ranging from low-risk to high-risk. The data from the patient’s IPI classifications was then used to create a simple Bayesian classifier. This classifier achieved an accuracy of 73.2% in predicting the mortality of DLBCL patients. Separately from the Bayesian classifier, several different types of “evolving fuzzy neural network” (EFuNN) classifiers were also developed to handle the genomic data. The best EFuNN classifier used a subset of 17 genes from the microarray data. This optimal EFuNN had an accuracy of 78.5%. The EFuNN classifier and the Bayesian classifier were then combined into a hierarchical modular system to generate a consensus prediction. This hybrid classifier attained an accuracy of 87.5%, a clear improvement over the performance of either classifier alone. This was also 10% better than the best performing machine learning classifier (77.6% by SVMs).

The EFuNN classifier was validated using a leave-one-out cross-validation strategy. This was likely due to the small sample size. As with Case Study #1, no external validation set was available to test the generality of the model. With only 56 patients (samples) being classified via 17 gene features, the sample per feature ratio (SFR) is just over 3. As a rule, an SFR of less than 5 does not necessarily guarantee a robust classifier (Somorjai et al. 2003). However, it is quite evident that the authors were aware of this issue and went to considerable lengths to justify their approach by explaining, in detail, the inner workings of their classifier. This included a description of how the Bayesian classifier was built, how the EFuNN works, and how the two classifiers work together to give a single prediction. In addition, the authors also investigated, and subsequently confirmed, the independence of the microarray data from the clinical data. This attention to detail is particularly exemplary for a machine learning investigation of this kind. This study nicely demonstrates how the power of using both clinical and genomic data in cancer prognosis can substantially enhance prediction accuracy.

### Case Study 3: Prediction of Cancer Recurrence

A total of 43% of the studies analyzed for this review applied machine learning towards the prediction of cancer relapse or recurrence. One particularly good example is the study of De Laurentiis et al. (1999), which actually addresses some of the drawbacks noted in the previous studies. These authors aimed to predict the probability of relapse over a 5 years period for breast cancer patients. A combination of 7 prognostic variables was used including clinical data such as patient age, tumor size, and number of axillary metastases. Protein biomarker information such as estrogen and progesterone receptor levels was also included. The aim of the study was to develop an automatic, quantitative prognostic method that was more reliable than the classical tumor-node-metastasis (TNM) staging system. TNM is a physician-based expert system that relies heavily on the subjective opinion of a pathologist or expert clinician. The authors employed an ANN-based model that used data from 2441 breast cancer patients (times 7 data points each) yielding a data set with more than 17,000 data points. This allowed the authors to maintain a sample-to-feature ratio of well over the suggested minimum of 5 (Somorjai et al. 2003). The entire data set was partitioned into three equal groups: training (1/3), monitoring (1/3), and test sets (1/3) for optimization and validation. In addition, the authors also obtained a separate set of 310 breast cancer patient samples from a different institution, for external validation. This allowed the authors to assess the generalizability of their model outside their institution—a process not done by the two previously discussed studies.

This study is particularly notable not only for the quantity of data and the thoroughness of validation, but also for the level of quality assurance applied to the data handling and processing. For instance, the data was separately entered and stored in a relational database and all of it was independently verified by the referring physicians to maintain quality. With 2441 patients and 17,000 data points in the data set, the sample size was sufficiently large that a normal population distribution of breast cancer patients could be assumed within the data set, even after partitioning. Regardless, the authors explicitly verified this assumption by looking at the distribution of the data for the patients within each set (training, monitoring, test, and external) and showed that the distributions were relatively similar. This quality assurance and attention to detail allowed the authors to develop a very accurate and robust classifier.

Since the aim of the study was to develop a model that predicted relapse of breast cancer better than the classical TNM staging system, it was important for the ANN model to be compared to TNM staging predictions. This was done by comparing the performance using a receiver operator characteristic (ROC) curve. The ANN model (0.726) was found to outperform the TNM system (0.677) as measured by the area under the ROC curve. Thi s study is an excellent example of a well-designed and well-tested application of machine learning. A sufficiently large data set was obtained and data for each sample was independently verified for quality assurance and accuracy. Furthermore, blinded sets for validation were available from both the original data set and from an external source to assess the generality of the machine learning model. Finally, the accuracy of the model was explicitly compared to that of a classical prognostic scheme, TNM staging. Perhaps the one drawback to this study was the fact that the authors only tested a single kind of machine learning (ANN) algorithm. Given the type and quantity of data used, it is quite possible that their ANN model may have been outperformed by another machine learning technique.

## Lessons, Limitations and Recommendations

The 3 case studies outlined in the preceding pages are just a few examples of how well-designed machine learning experiments should be conducted and how the methods and results should be described, validated and assessed – especially in cancer prediction and prognosis. There are obviously many other examples of equally good studies with equally impressive results (see Table 2). However, it is also important to note that not all machine learning studies are conducted with the same rigor or attention to detail as with these case studies. Being able to identify potential problems in either the experimental design, validation or learner implementation is critical not only for those wishing to use machine learning, but also for those needing to evaluate different studies or to assess different machine learning options.

One of the most common problems seen among the studies surveyed in this review was the lack of attention paid to data size and learner validation. In other words, there are a number of studies with sloppy experimental design. A minimum requirement for any machine learning exercise is having a sufficiently large data set that can be partitioned into disjoint training and test sets or subjected to some reasonable form of n-fold cross-validation for smaller data sets. Typically 5-fold (iteratively taking 20% of the training data out to serve as testing data) or 10-fold cross-validation (iteratively taking 10% of the training data out to serve as testing data) is sufficient to validate most any learning algorithm. This kind of rigorous internal validation is critical to creating a robust learner that can consistently handle novel data. Beyond the standard practice of internal validation, it is particularly beneficial to perform a validation test using an external data source. External validation is an important “sanity” check and it also helps to catch or minimize any bias that may be imposed by site or person-specific clinical measurement practices. Of course, this external validation set must also be of sufficiently large size to ensure reproducibility.

As has been frequently noted before, the size of a given training set has several implications pertaining to robustness, reproducibility and accuracy. The first implication is that for a smaller sample size, almost any model is prone to overtraining. Overtraining can lead to reported accuracies that may be misleading or erroneous. For instance, one early study reported only a single misclassification during the training and testing of an ANN for predicting the survival of hepatectomized patients using 9 separate features (Hamamoto et al. 1995). However, the entire data set (training and testing) consisted of just 58 patients. This particular study then used an external data set to validate the model where the authors prospectively predicted the survival outcome with 100% accuracy.

However, the external test set only consisted of 11 patients. The fact that 100% accuracy is attained for a prospective prediction is impressive, but given the size of the validation set and the small sample-per-feature ratio, some doubt may be cast on the robustness of the predictor. Certainly a larger validation set would be desirable to reinforce the claim of 100% accuracy. In another example, only 28 cases were used to build an ANN for predicting throat cancer recurrence that made use of the expression levels of 60 genes from microarray data (Kan et al. 2004). The accuracy of the model was claimed to be 86%, but this is particularly suspect given the very small sample size. Indeed it is quite likely that this ANN was over-trained.

The size of a given data set also significantly affects the sample-per-feature ratio. As a rule, the sample-per-feature ratio should be at least 5–10 (Somorjai et al. 2003). Small sample-per-feature ratios are a particularly big problem for microarray studies, which often have thousands of genes (ie features), but only hundreds of samples. The study by Ohira et al. (2005) provides one such example of the problems one may encounter trying to process too much microarray data. These authors created a probabilistic output statistical classifier to predict prognosis of neuroblastoma patients using microarray data from 136 tumor samples. Each microarray had 5340 genes, leading to a sample-per-feature ratio of ~0.025. A sample-per-feature ratio this small is highly susceptible to the problems of overtraining. Furthermore, with a sample-per-feature ratio of this size it is also possible to develop highly redundant classification models which perform equally well despite being trained on different subsets of genes. The problem with redundant models is that the robustness of any one model cannot be guaranteed as more test cases become available.

Data size is not the only limitation for effective machine learning. Data set quality and careful feature selection are also equally important (recall: “garbage in=garbage out”). For large data sets data entry and data verification are of paramount importance. Often careless data entry can lead to simple off-by-one errors in which all the values for a particular variable are shifted up or down by one row in a table. This is why independent verification by a second data-entry curator or data checker is always beneficial. Further verification or spot checking of data integrity by a knowledgeable expert, not just a data entry clerk, is also a valuable exercise. Unfortunately, the methods employed to ensure data quality and integrity are rarely discussed in most machine learning papers.

Just as data quality is important so too is feature quality. Certainly the subset of features chosen to train a model could mean the difference between a robust, accurate model and one that is flawed and inaccurate. Ideally features should be chosen that are reproducible and precisely measurable from one lab (or clinic) to the next. One study (Delen et al. 2005) used “primary site code” and “site specific surgery code” as features to predict breast cancer survivability. While these clinical features may be helpful in determining the outcome for breast cancer patients at this particular hospital, for this moment in time, they may become irrelevant overtime. Even worse, if new site codes or site specific surgery codes are created, the model will have to be re-trained to account for the new codes. Similar feature selection problems often occur with histological assessments. As good as many pathologists are there is always some inconsistency (up to 30% in many cases) between different histopathological assessments from different sites or different pathologists. As a rule, the best features are those that are highly reproducible, universal or absolute (age, gender, weight, certain biomarker measurements, etc). Even with these seemingly robust features it is important to remember that clinical data sets are not static entities. With time the importance or relevance of these clinical measures may evolve over time with some features being added, modified or deleted. Therefore a classifier must also be able to adapt to different feature sets over time too.

Another important lesson that was learned from assessing many of these machine learning papers was the value of using multiple predictor models based on different machine learning techniques. While ANNs are often considered to be very sophisticated and advanced machine learning methods, ANNs are not always the best tools for the job. Sometimes simpler machine learning methods, like the naïve Bayes and decision tree methods can substantially outperform ANNs (Delen et al. 2005). Assessing the performance of a machine learning predictor against other predictors is critical to choosing the optimal tool. It is also critical to deciding if the method is any better than previously existing schemes. Ideally, any newly published machine learning model should be compared against either another kind of learning model, a traditional statistical model or an expert-based prognostic scheme such as the TNM staging system. As seen in Table 2, sometimes the more sophisticated machine learning methods do not lead to the best predictors. In some cases, traditional statistics actually outperform machine learning methods (Kaiserman et al. 2005; Kim et al. 2003). Unfortunately, only about 17% of the papers reviewed here tested more than one machine learning classifier.

It is also important to remember that the machine learning process is essentially a computational experiment. Like any experiment it is based on a hypothesis, it follows defined procedures and it requires data to be validated. Because machine learners represent true experimental procedures, they should be treated as such. Therefore detailed methodological documentation is of paramount importance. Ideally, the data sets used for training and testing should be described in detail and made available to the public. Information about training and testing data should also be well-described including the way in which the sets were partitioned. Likewise the details regarding the algorithms used and their implementations should be provided or recorded to permit others to verify and reproduce the results. In principle, the results from a good machine learning experiment should be as reproducible as any other standard lab protocol.

## Conclusion

In this review we have attempted to explain, compare and assess the performance of different machine learning that are being applied to cancer prediction and prognosis. Specifically we identified a number of trends with respect to the types of machine learning methods being used, the types of training data being integrated, the kinds of endpoint predictions being made, the types of cancers being studied and the overall performance of these methods in predicting cancer susceptibility or outcomes. While ANNs still predominate it is evident that a growing variety of alternate machine learning strategies are being used and that they are being applied to many types of cancers to predict at least three different kinds of outcomes. It is also clear that machine learning methods generally improve the performance or predictive accuracy of most prognoses, especially when compared to conventional statistical or expert-based systems. While most studies are generally well constructed and reasonably well validated, certainly greater attention to experimental design and implementation appears to be warranted, especially with respect to the quantity and quality of biological data. Improvements in experimental design along with improved biological validation would no doubt enhance the overall quality, generality and reproducibility of many machine-based classifiers. Overall, we believe that if the quality of studies continues to improve, it is likely that the use of machine learning classifier will become much more commonplace in many clinical and hospital settings.

## Figures and Tables

**Table 1.** Summary of benefits, assumptions and limitations of different machine learning algorithms

Machine Learning Algorithm	Benefits	Assumptions and/or Limitations
Decision Tree (Quinlan 1986)	easy to understand and efficient training algorithm order of training instances has no effect on training pruning can deal with the problem of overfitting	classes must be mutually exclusive final decision tree dependent upon order of attribute selection errors in training set can result in overly complex decision trees missing values for an attribute make it unclear about which branch to take when that attribute is tested
Naïve Bayes (Langley et al 1992)	foundation based on statistical modelling easy to understand and efficient training algorithm order of training instances has no effect on training useful across multiple domains	assumes attributes are statistically independent* assumes normal distribution on numeric attributes classes must be mutually exclusive redundant attributes mislead classification attribute and class frequencies affect accuracy
*k*-Nearest Neighbour (Patrick & Fischer 1970; Aha 1992)	fast classification of instances useful for non-linear classification problems robust with respect to irrelevant or novel attributes tolerant of noisy instances or instances with missing attribute values can be used for both regression and classification	slower to update concept description assumes that instances with similar attributes will have similar classifications assumes that attributes will be equally relevant too computationally complex as number of attributes increases
Neural Network (Rummelhart et al 1986)	can be used for classification or regression able to represent Boolean functions (AND, OR, NOT) tolerant of noisy inputs instances can be classified by more than one output	difficult to understand structure of algorithm too many attributes can result in overfitting optimal network structure can only be determined by experimentation
Support Vector Machine (Vapnik 1982; Russell and Norvig, p 749–52)	models nonlinear class boundaries overfitting is unlikely to occur computational complexity reduced to quadratic optimization problem easy to control complexity of decision rule and frequency of error	training is slow compared to Bayes and Decision Trees difficult to determine optimal parameters when training data is not linearly separable difficult to understand structure of algorithm
Genetic Algorithm (Holland 1975)	simple algorithm, easy to implement can be used in feature classification and feature selection primarily used in optimization always finds a “good” solution (not always the best solution)	computation ordevelopment of scoring function is non trivial not the most efficient method to find some optima, tends to find local optima rather than global complications involved in the representation of training/output data

**Table 2:** Survey of machine learning methods used in cancer prediction showing the types of cancer, clinical endpoints, choice of algorithm, performance and type of training data.

Cancer Type	Clinical Endpoint	Machine Learning Algorithm	Benchmark	Improvement (%)	Training Data	Reference
bladder	recurrence	fuzzy logic	statistics	16	mixed	Catto et al, 2003
bladder	recurrence	ANN	N/A	N/A	clinical	Fujikawa et al, 2003
bladder	survivability	ANN	N/A	N/A	clinical	Ji et al, 2003
bladder	recurrence	ANN	N/A	N/A	clinical	Spyridonos et al, 2002
brain	survivability	ANN	statistics	N/A	genomic	Wei et al, 2004
breast	recurrence	clustering	statistics	N/A	mixed	Dai et al, 2005
breast	survivability	decision tree	statistics	4	clinical	Delen et al, 2005
breast	susceptibility	SVM	random	19	genomic	Listgarten et al, 2004
breast	recurrence	ANN	N/A	N/A	clinical	Mattfeldt et al, 2004
breast	recurrence	ANN	N/A	N/A	mixed	Ripley et al, 2004
breast	recurrence	ANN	statistics	1	clinical	Jerez-Aragones et al, 2003
breast	survivability	ANN	statistics	N/A	clinical	Lisboa et al, 2003
breast	treatment response	ANN	N/A	N/A	proteomic	Mian et al, 2003
breast	survivability	clustering	statistics	0	clinical	Seker et al, 2003
breast	survivability	fuzzy logic	statistics	N/A	proteomic	Seker et al, 2002
breast	survivability	SVM	N/A	N/A	clinical	Lee et al, 2000
breast	recurrence	ANN	expert	5	mixed	De Laurentiis et al, 1999
breast	survivability	ANN	statistics	1	clinical	Lundin et al, 1999
breast	recurrence	ANN	statistics	23	mixed	Marchevsky et al, 1999
breast	recurrence	ANN	N/A	N/A	clinical	Naguib et al, 1999
breast	survivability	ANN	N/A	N/A	clinical	Street, 1998
breast	survivability	ANN	expert	5	clinical	Burke et al, 1997
breast	recurrence	ANN	statistics	N/A	mixed	Mariani et al, 1997
breast	recurrence	ANN	expert	10	clinical	Naguib et al, 1997
cervical	survivability	ANN	N/A	N/A	mixed	Ochi et al, 2002
colorectal	recurrence	ANN	statistics	12	clinical	Grumett et al, 2003
colorectal	survivability	ANN	statistics	9	clinical	Snow et al, 2001
colorectal	survivability	clustering	N/A	N/A	clinical	Hamilton et al, 1999
colorectal	recurrence	ANN	statistics	9	mixed	Singson et al, 1999
colorectal	survivability	ANN	expert	11	clinical	Bottaci et al, 1997
esophageal	treatment response	SVM	N/A	N/A	proteomic	Hayashida et al, 2005
esophageal	survivability	ANN	statistics	3	clinical	Sato et al, 2005
leukemia	recurrence	decision tree	N/A	N/A	proteomic	Masic et al, 1998
liver	recurrence	ANN	statistics	25	genomic	Rodriguez-Luna et al, 2005
liver	recurrence	SVM	N/A	N/A	genomic	Iizuka et al, 2003
liver	susceptibility	ANN	statistics	–2	clinical	Kim et al, 2003
liver	survivability	ANN	N/A	N/A	clinical	Hamamoto et al, 1995
lung	survivability	ANN	N/A	N/A	clinical	Santos-Garcia et al, 2004
lung	survivability	ANN	statistics	9	mixed	Hanai et al, 2003
lung	survivability	ANN	N/A	N/A	mixed	Hsia et al, 2003
lung	survivability	ANN	statistics	N/A	mixed	Marchevsky et al, 1998
lung	survivability	ANN	N/A	N/A	clinical	Jefferson et al, 1997
lymphoma	survivability	ANN	statistics	22	genomic	Ando et al, 2003
lymphoma	survivability	ANN	expert	10	mixed	Futschik et al, 2003
lymphoma	survivability	ANN	N/A	N/A	genomic	O’Neill and Song, 2003
lymphoma	survivability	ANN	expert	N/A	genomic	Ando et al, 2002
lymphoma	survivability	clustering	N/A	N/A	genomic	Shipp et al, 2002
head/neck	survivability	ANN	statistics	11	clinical	Bryce et al, 1998
neck	treatment response	ANN	N/A	N/A	clinical	Drago et al, 2002
ocular	survivability	SVM	N/A	N/A	genomic	Ehlers and Harbour, 2005
osteosarcoma	treatment response	SVM	N/A	N/A	genomic	Man et al, 2005
pleural mesothelioma	survivability	clustering	N/A	N/A	genomic	Pass et al, 2004
prostate	treatment response	ANN	N/A	N/A	mixed	Michael et al, 2005
prostate	recurrence	ANN	statistics	0	clinical	Porter et al, 2005
prostate	treatment response	ANN	N/A	N/A	clinical	Gulliford et al, 2004
prostate	recurrence	ANN	statistics	16	mixed	Poulakis et al, 2004a
prostate	recurrence	ANN	statistics	11	mixed	Poulakis et al, 2004b
prostate	recurrence	SVM	statistics	6	clinical	Teverovskiy et al, 2004
prostate	recurrence	ANN	statistics	0	clinical	Kattan, 2003
prostate	recurrence	genetic algorithm	N/A	N/A	mixed	Tewari et al, 2001
prostate	recurrence	ANN	statistics	0	clinical	Ziada et al, 2001
prostate	susceptibility	decision tree	N/A	N/A	clinical	Crawford et al, 2000
prostate	recurrence	ANN	statistics	13	clinical	Han et al, 2000
prostate	treatment response	ANN	N/A	N/A	proteomic	Murphy et al, 2000
prostate	recurrence	naïve Bayes	statistics	1	clinical	Zupan et al, 2000
prostate	recurrence	ANN	N/A	N/A	clinical	Mattfeldt et al, 1999
prostate	recurrence	ANN	statistics	17	clinical	Potter et al, 1999
prostate	recurrence	ANN	N/A	N/A	mixed	Naguib et al, 1998
skin	survivability	ANN	expert	14	clinical	Kaiserman et al, 2005
skin	recurrence	ANN	expert	27	proteomic	Mian et al, 2005
skin	survivability	ANN	expert	0	clinical	Taktak et al, 2004
skin	survivability	genetic algorithm	N/A	N/A	clinical	Sierra and Larranga, 1998
stomach	recurrence	ANN	expert	28	clinical	Bollschweiler et al, 2004
throat	recurrence	fuzzy logic	N/A	N/A	clinical	Nagata et al, 2005
throat	recurrence	ANN	statistics	0	genomic	Kan et al, 2004
throat	survivability	decision tree	statistics	N/A	proteomic	Seiwerth et al, 2000
thoracic	treatment response	ANN	N/A	N/A	clinical	Su et al, 2005
thyroid	survivability	decision tree	statistics	N/A	clinical	Kukar et al, 1997
tropho-	survivability	genetic algorithm	N/A	N/A	clinical	Marvin et al, blastic 1999
